# Advanced multi-objective trajectory planning for robotic arms using a multi-strategy enhanced NSGA-II algorithm

**DOI:** 10.1371/journal.pone.0324567

**Published:** 2025-05-29

**Authors:** Yanqin Fan, Yinan Peng, Jianlin Liu

**Affiliations:** 1 School of Science, Guilin University of Aerospace Technology, Guilin, China; 2 Shanghai Palmim Information Technology Ltd., Shanghai, China; 3 Key Laboratory of Advanced Manufacturing Technology, Ministry of Education, Guizhou University, Gui-yang, Guizhou, China; G H Raisoni College of Engineering and Management, Pune, INDIA

## Abstract

Facing the problems of large-scale rapid and disorderly loading, the robotic arm has the problems of large start-stop impact, easy to shake, and reduced production efficiency and service life, this paper proposes a robotic arm motion planning method based on the improved multi-objective algorithm called LNSGA-II. Firstly, the artificial potential field method is used to plan the shortest path without collision, extract the key motion sequences, and establish the multi-objective function to improve the operating efficiency of the robotic arm, the smoothness of the motion trajectory, and the reduction of energy consumption. Then to solve the nonlinear constraints in the multi-objective trajectory planning, the infeasibility degree is designed, and the NSGA-II is improved by using the mutation chaos strategy and the dynamic goal-oriented development strategy. Numerical and trajectory planning experiments are conducted successively with the remaining five well-known multi-objective algorithms, and the experimental results demonstrate the superiority of LNSGA-II. Finally, the digital twin platform of MATLAB-CoppeliaSim-UR16e verifies the effectiveness of the method in real grasping tasks.

## 1. Introduction

The rapid development of technologies such as the Internet of Things, cloud computing, big data, and mobile Internet has sparked a new wave of industrial revolution, which highlights the critical importance of the manufacturing industry [1–4]. Lately, many countries have formulated and implemented their respective strategies, including the European Union’s “Industry 5.0: A Vision for Change in Europe” released in early 2022, the German “National Industrial Strategy 2030” and China’s “Made in China 2025” [5,6]. Along with the trend of the industrial revolution, the basic industries around the world are developing rapidly, and robotic arms, as a kind of highly intelligent, integrated and automated machine, are leading the way in the manufacturing industry [7–9]. In actual production tasks, factory personnel usually set the path between the material and the operating table to control the robotic arm to grasp the goal. In the grasping path, only the three-dimensional coordinates of the manipulator work domain, the material and the operating table are often involved, and the completion of the grasping action is only considered, without involving the compliance of the servo motor driven manipulator. However, in the case of large-scale rapid and disorderly feeding, the continuous high-intensity gripping and transportation of materials by the robotic arm can lead to significant start-stop impact, causing jitter, reducing production efficiency and service life, and potentially damaging the servo motor. In the gripping process of the robotic arm, the necessary trajectory planning can make the movement curve of the robotic arm joints like angular displacement, angular velocity, angular acceleration and so on smoother and continuous, and at the same time, it can also protect the servo motor [10,11].

Trajectory planning for robotic arms motion is usually done using polynomial planning [12,13] and spline curve planning methods [14]. Yongzheng Cong [15] utilized straight line trajectory interpolation and circular arc interpolation methods for Cartesian space-based trajectory planning for a two-arm robot and validated it within a ROS system, proving that the methods are feasible. Xiangfei Li [16] proposed a robot position and attitude synchronization method with third-order NURBS curves, which was simulated using blade-type and fan-type robots, and the results showed that this method was able to satisfy the given constraints. With the continuous improvement and development of modern industrial technology, the production application scenarios of robotic arms have become more and more extensive and in-depth. Under a number of complicated working conditions and realistic needs, single-objective trajectory planning algorithms have been unable to meet the diverse, complex and precise requirements of the production operations of robotic arms.

Aiming at the multi-objective problem of fast and smooth operation of segmented assembly robots in non-circular shield machines, Sun [17] proposed a method based on B-spline interpolation and non-dominated sorting evolutionary algorithm. The experimental results show that the high-order B-spline curve of this method obtains a lower acceleration peak and a smoother global trajectory, and achieves the optimal trade-off of multi-objective trajectory planning. In order to optimize the crane lifting path planning problem in industrial lifting, Panpan Cai [18] et al. formulated it as a multi-objective nonlinear integer optimization problem with implicit constraints and designed a master-slave parallel genetic algorithm, and the experimental results showed that this method can efficiently generate high-quality lifting paths in complex environments. Wang [19] proposed an optimal trajectory planning method based on coupling interpolation function selection, which took the task time and impact of industrial robots as optimization objectives. Under the background of robot stirring operation in the process of automatic block casting loading, the proposed method is used to derive the optimal trajectory of time and jitter. The experimental results show the effectiveness of the proposed method. In multi-objective trajectory planning for robotic arms, researchers mainly use linear weighting to convert the multi-objective function into single-objective function for solving. Due to the different magnitudes between different objectives, it is not possible to accurately assign the weights to ensure the effective continuity of the joint acceleration and jerk, potentially resulting in damage to the high-speed working robotic arm. Although all these methods can achieve the purpose of trajectory planning, their effectiveness is limited in multi-objective optimization [20,21].

With the continuous development of intelligent manufacturing, robotic arms gradually replace humans to complete repetitive, dangerous or high-precision work, improving production efficiency and safety [22]. Concurrently, when the structure and function of the manipulator become more and more complex, the challenges associated with data collection and processing have also grown. But the emergence of digital twins effectively solves this problem. Digital twin accelerates the digital transformation of robotic arms, and the twin model can provide the verification of the feasibility of various schemes, which has great potential and role in the production and assembly process [23,24].

Aiming at the problems of large start-stop impact, easy jitter, low production efficiency and service life of the manipulator when feeding large-scale, fast and disorderly, this paper delineates the paths between end-effector, materials and obstacles through artificial potential field (APF) [25,26] regulations. According to the passing time and order of the key points along the motion path, a multi-objective function is established to improve the operation efficiency of the robot, the smoothness of the running trajectory and the reduction of the energy consumption of the robot. The LNSGA-II is used to solve the problem, and the 7-order B-splines curve is used to complete the motion trajectory planning in the joint space of the manipulator. The section 2 of this paper is the mathematical modeling of robotic arm multi-objective trajectory planning. In Section 3, a new multi-objective algorithm called LNSGA-II is proposed. In Section 4, the experiment is set up, which includes the CEC2009 test set experiments and the robotic arm multi-objective trajectory planning experiments. The Section 5 is the manipulator grasping practice based on digital twin and the Section 6 is the summary. The main contributions of this study are summarized as follows:

(1) After path planning, a multi-objective function incorporating efficiency, energy consumption, and impact is formulated based on the passing time and sequence of key points, and a mathematical model for manipulator trajectory planning is established.
(2) To address issues of uneven initial individual randomization and sensitivity to iteration numbers in NSGA-II, a mutation chaos strategy and dynamic goal-oriented strategy are introduced, forming an improved LNSGA-II. The infeasibility metric is also designed to handle nonlinear constraints, and LNSGA-II is ultimately used to optimize the 7th-order B-spline curve for multi-objective trajectory planning.
(3) Experiments on the CEC2009 test set show that LNSGA-II surpasses the original algorithm in convergence speed and optimization performance, demonstrating strong competitiveness compared to five other multi-objective optimization algorithms.
(4) A digital twin platform is constructed using MATLAB-CoppeliaSim-UR16e, applying the proposed trajectory planning model to the robotic arm’s actual grasping tasks, verifying its effectiveness.

Section 2 presents the development of the multi-objective trajectory planning model for the manipulator. Section 3 reviews the original NSGA-II algorithm and introduces the motivation and strategies behind the improvement of the LNSGA-II algorithm. Section 4 discusses the experimental results, including numerical experiments using the CEC2009 benchmark and manipulator trajectory planning results. Section 5 introduces the digital twin platform based on MATLAB-CoppeliaSim-UR16e, where the digital twin framework and trajectory planning model are validated through a real grasping task. Section 6 concludes the paper.

## 2. Multi-objective trajectory planning modeling of the robotic arm

### 2.1 Motion path of the robotic arm

Motion planning [27] mainly studies the construction of a collision-free and efficient motion sequence of a robotic arm in the workspace from the start point to the end. Robotic arm motion planning is usually subdivided into two levels: path planning [28,29] and trajectory planning [30,31].

**Fig 1:**
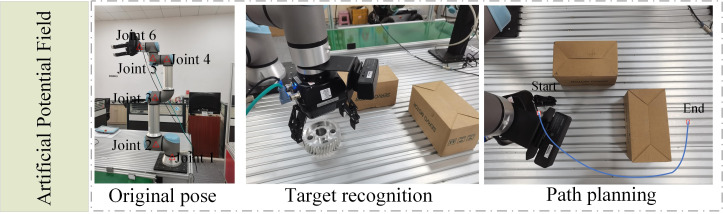
Path planning for the 6-DOF robotic arm.

In the actual operation space, there are often obstacles between the material and the goal point. Through the necessary path planning, the manipulator can grasp the material more quickly and accurately. In this study, the APF will be used for path planning of the robotic arm, and a shortest collision-free path will be designed, as shown in Fig 1, which will pave the way for subsequent trajectory planning.

Aiming at the actual operation task of the manipulator on the target, the path between the end-effector and the target is planned in the actual space. According to the passing time and sequence of the key points of the motion path, the joint trajectory planning of the manipulator is carried out by fully considering the path constraints and joint constraints. In the Table 1, *q* is the joint position.

**Table 1:** The joint position sequence of robotic arms.

Joint position	*q*/(°)					
	1	2	3	4	5	6
Pos_0_	-20.17	-8.05	89.70	8.39	-90.05	-69.88
Pos_1_	-34.62	-4.67	104.30	-9.58	-90.05	-55.43
Pos_2_	-37.27	0.75	100.92	-11.62	-90.05	-52.77
Pos_3_	-49.45	8.65	97.50	-16.10	-90.05	-40.60
Pos_4_	-76.38	8.06	97.15	-15.17	-90.05	-13.67
Pos_5_	-80.19	13.02	89.92	-12.89	-90.05	-9.86
Pos_6_	-93.38	14.49	81.42	-5.87	-90.05	3.33
Pos_7_	-109.56	5.76	77.03	7.26	-90.05	19.51

### 2.2 Multi-objective trajectory planning modeling

When the robotic arm goes from the start point to the end in the task space, a series of key points are obtained by inverse kinematics calculation, $Qsm=\{(qx\alpha a,t_{s}),(m=1,2\ldots \;,\;s=0,1\ldots n\}$, *m* is the number of robotic arm joints and *s* is the key point, fm(t)=qsms is obtained through the robotic arm trajectory planning. The trajectory of the robotic arm motion is constructed using k-times B-splines curve [32].


$$B(t)=\underset{j=0}{\overset{n}{\sum }}\;d_{j}F_{j,k}(t)$$ (1)

Where $d_{j}$ is the coordinates of the control point, $F_{j,k}(t)$ is the k-times B-spline basis function. B(t) refers to the joint position at moment t, which is defined by the control point $d_{j}$ and the node vector $T=[t_{0},t_{1},...,t_{n+2k}]$ together, and the canonical definition domain: $t_{0}=t_{1}=...=t_{k}=0,t_{n+k}=t_{n+k+1}=...=t_{n+2k}=1$. By inverting the control vertices of the B-spline curve, so that each robotic arm joint passes through the key point qsms. Additionally, the segmented connection points of the trajectory curve correspond to the key nodes, with the starting point and end point being the same. The time node $t_{j}$ is normalized according to the cumulative chord length parameterization method to obtain the inner node value.


$$\begin{array}{c} \begin{array}{cc} & t_{p}=t_{p-1}+\frac{\Delta t_{p-k-1}}{\sum_{j=0}^{n-1}\Delta t_{j}},p=k+1,...n+k-1\\ & \Delta t_{j}=t_{j+1}-t_{j}(j=0,1,...,n-1) \end{array} \end{array}$$ (2)

Thus, n-1 equations satisfying the interpolation conditions are obtained as follows.


B(tp)=∑i+kj=idjFj,k(tp)=QSmtp∈[tk,tk+n] (3)

Therefore k-1 additional equations need to be added for solving. Assuming that a 7th-order B-splines curve is used with inputs of velocity, acceleration and jerk at the start and end points, the additional equations are as follows:


$$\left\{\begin{array}{l} B^{\prime }(t_{7})=v_{start},B^{\prime }(t_{7+n})=v_{end},\\ B^{\prime \prime }(t_{7})=a_{start},B^{\prime \prime }(t_{7+n})=a_{end},\\ B^{\prime \prime \prime }(t_{7})=j_{start},B^{\prime \prime \prime }(t_{7+n})=j_{end}. \end{array}\right.$$ (4)

Where, $B^{\prime }(t),\;B^{\prime \prime }(t),\;B^{\prime \prime \prime }(t)$ are the 1 ~ 3 derivatives of the B-spline curve respectively, and are the velocity, acceleration and jerk of each joint in turn. The derivatives of each order can be derived according to the de Boer recurrence formula:


$$\begin{array}{c} \begin{array}{cc} & B^{r}(t)=\underset{j=p-k+r}{\overset{p}{\sum }}\;d_{j}^{r}F_{j,k-r}(t),t_{p}\leq t<t_{p+1}\\ & d_{j}^{r}=\left\{ \begin{array}{cc} \;\;\;\;\;\;\;\;\;\;\;\;\;\;\;\;\;\;\;\;\;\;\;\;\;\;\;\;\;\;\;\;\;\;\;\;\;\;\;\;\;d_{j}, & \;\;\;\;\;\;\;\;\;\;r=0\\ (k+1-r)\frac{d_{j}^{r-1}-d_{j-1}^{r-1}}{t_{j+k+1-r}-t_{j}}, & r=1,2...\\ j=p-k+r & \end{array}\right. \end{array} \end{array}$$ (5)

*r* is the order of derivation and $d_{j}^{r}$ denotes the formula for the rth order derivative of the jth control node, which in turn yields n + k linear equations. By solving the equations, the control vertex vectors of the B-spline trajectory curves for each joint are obtained. Based on the normalized time, the position *q*_*s*_ passed by each joint at the moment *t*_*s*_ can be obtained. The trajectory kinema*t*ic constraints are converted into constraints on the B-spline control points as follows:


$$\begin{array}{c} \left\{ \;\;\;\;\;\;\;\;\;\begin{array}{cc} & {\left|d_{mj}^{1}\right|}_{\max}\leq v_{m\max},j=1,2...n+k-1\\ & {\left|d_{mj}^{2}\right|}_{\max}\leq a_{m\max},j=1,2...n+k-1\\ & {\left|d_{mj}^{3}\right|}_{\max}\leq j_{m\max},j=1,2...n+k-1 \end{array}\right. \end{array}$$ (6)

where, $d_{mj}^{1},d_{mj}^{2},d_{mj}^{3}$ denotes the jth control vertex of the mth joint of the velocity, acceleration, and jerk trajectory curve, respectively. $f_{1}$ is the movement time, which measures the efficiency of the robotic arm operation. $f_{2}$ is the average acceleration, measuring the energy consumption of the robotic arm. $f_{3}$ is joint jerk, which measures the smoothness of manipulator motion. It is the objective function with optimal efficiency, optimal energy consumption, and optimal impact.


min  f1=∑s=0sΔt=∑s=0s(ts+1−ts)        f2=∑m=1m1T∫0Ta2mdt=∑m=1m1T∫0T(q⬝⬝)2dt       f3=∑m=1m1T∫0TJ2mdt=∑m=1m1T∫0T(q⬝⬝⬝)2dt (7)

With $\Delta t_{k}=t_{k+1}-t_{k}$ as the optimization variable, the ‘efficiency-energy-impact’ objective function is established under the above constraints, and the feasible solution is found by the multi-objective optimization algorithm. In order to verify the real-time performance of the algorithm, the time spent on the B-spline trajectories of different orders is tested, and the time spent is 27.785s, 53.1529s, and 78.4184s for 10^5^ operations on 3, 5, and 7 B-spline values respectively under the same test platform, and the algorithm times are all in the same order of magnitude (10^-5^s), so that the increase in order has little effect on the real-time performance of the system.

## 3. Multi-strategy improved LNSGA-II algorithm

### 3.1 Original NSGA-II algorithm

A fast and Elitist Multi-objective Genetic Algorithm (NSGA-II) was first published by Kalyanmoy Deb in 2002 [33]. NSGA-II involves three key stages: non-dominated sorting, crowding degree calculation, elite selection iteration and crossover and mutation.

#### 3.1.1 Non-dominated sorting.

Find the non-dominated optimal solution of the current population and assign rank 1. The individuals of rank 1 are removed from the population, and new non-dominated solutions are found in the remaining individuals, which are assigned rank 2. The non-dominated sorting process of the population is repeated until all individuals are set corresponding ranks and their crowding distances are calculated.

#### 3.1.2 Elite selection.

The selection process makes the optimization towards the Pareto optimal solution and makes the solution uniformly dispersed. After sorting and crowding distance calculation, each individual i in the population gets two attributes, the non-dominated ordinal number i_rank_ and the crowding distance i_d_. The round-robin selection operator is used, that is, two individuals are randomly selected. When i_rank_ < j_rank_ or i_rank_ = j_rank_ and i_d_ > j_d_, i individual is better than j individual. If the non-dominated ordering of the two individuals is different, take the individual with the lower order number; if the two individuals are at the same level, take the individual with the surrounding i_d_ > j_d_. Where j_d_ and j_rank_ are denoted as the nondominant ordinal number and crowding distance of the jth population individual.

#### 3.1.3 Crossover and mutation.

Crossover operation is performed on the parent individual, that is, for a given random crossover point, the parts of the two parent individuals on both sides of the crossover point are exchanged. All the individuals of the parent P_t_ and the offspring Q_t_ are synthesized into a unified population R_t_, and the number of individuals of R_t_ is 2N. The population R_t_ is quickly non-dominated sorted and the local crowding distance of each individual is calculated. Individuals are selected one by one according to the level of hierarchy. When the number of individuals reaches N, a new parent population P_t+1_ is formed. On the basis of the new parent population P_t+1_, a new round of selection, crossover and mutation is started to form a new offspring population Q_t+1_.

The multi-objective trajectory planning problem of manipulator joints is essentially a constraint problem. In order to speed up the efficiency of NSGA-II, this paper first designs an infeasible degree to deal with multi-objective constraint problems. Furthermore, due to the random generation of initial individuals in NSGA-II, a common issue emerges where individuals cluster in specific regions, leading to limited population diversity. Therefore, this paper proposes an innovative mutation chaos strategy for population initialization. Lastly, addressing the sensitivity of NSGA-II to the iteration count, under limitless computation time, NSGA-II consistently converges to the optimal solution. This paper proposes a dynamic goal-oriented development to expedite individual development towards the target solution in the later iteration phase.

### 3.2 Proposed LNSGA-II algorithm

#### 3.2.1 Design of infeasibility.

In dealing with constraint problems, the conventional method is to transform the transboundary solution into the boundary extreme point. This paper designs a kind of infeasibility degree of the target solution to address the constraint, which is defined as the sum of squares of all conflicting constraint values.


$$\psi (x_{i})=\underset{a=1}{\overset{A}{\sum }}\;{\left[G_{a}(x_{i})\right]}^{2}+\underset{b=1}{\overset{B}{\sum }}\;{\left[\min\{0,T_{b}(x_{i}\}\right]}^{2}$$ (8)

Here, *a* and *b* represent the count of equality and inequality constraints, respectively. The infeasible degree refers to the distance between the current solution and the feasible region. A solution *x*_*i*_ is considered feasible when its infeasibility degree is 0. The larger the infeasible degree is, the farther *x*_*i*_ is from the feasible region. Additionally, an infeasible threshold is set to determine whether *x*_*i*_ is accepted for the next genetic operation.


$$\begin{array}{c} \begin{array}{cc} & \psi_{\text{th}}=\frac{1}{\omega }\underset{i=1}{\overset{N_{\text{pop}}}{\sum }}\;\frac{\psi (x_{i})}{N_{\text{pop}}}\\ & \omega =\frac{Iter}{Max-iter} \end{array} \end{array}$$ (9)

Here, ω is the annealing factor, which increases with the increase of the number of iterations. Iter and Max-iter represent the current and maximum iterations, correspondingly. *N*_*pop*_ is the population size. When the infeasibility of the current solution is less than the threshold, the solution is accepted, otherwise it is rejected. To maintain a consistent population size, any rejected solutions are substituted with the solution with the lowest infeasibility.

#### 3.2.2 Mutation chaos strategy.

Random generation of NSGA-II individuals during the initial stage results in limited population diversity, significant boundary clustering, and reduced individual variability. Chaos is mathematically defined as the randomness generated by a simple deterministic system [34,35]. Due to the ergodicity of chaos, chaos-based mapping is used to solve the problem of premature convergence of optimization algorithms [36–39]. For the present, it is often used by scholars as Logistic mapping, Sinusoidal mapping, etc., and the specific expressions of the above two chaotic mappings are shown in Eq. 10. Where *a* and *μ* are the system parameters, when *a* = 4, the Logistic chaotic map shows complete chaotic behavior within [0, 1]. Similarly, when the value of *μ* is equal to 1, the dynamics of the sinusoidal chaotic map shows complex and chaotic characteristics.


$$\begin{array}{cc} & \beta_{t}=a\cdot \beta_{t-1}\cdot (1-\beta_{t-1})\\ & \sigma_{t}=\mu \cdot \sin(\pi \sigma_{t-1}) \end{array}$$ (10)

**Fig 2:**
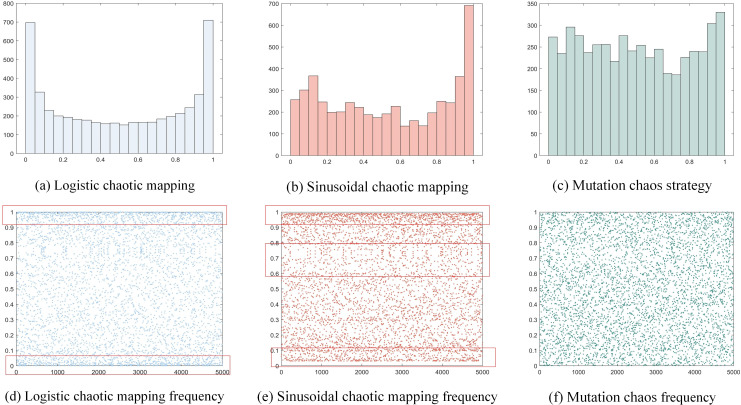
Sinusoidal chaotic map and its variant.


The histograms and frequency plots of the Logistic chaotic mapping and Sinusoidal chaotic mapping after 5000 cycles are displayed in Fig 2. From Fig 2a, the histogram of the Logistic mapping shows a distribution pattern with higher at both ends and lower in the middle. The frequencies between [0, 0.02] and [0.95, 1] are as high as 700, while the frequencies between [0.1, 0.9] are only around 200, indicating that in this mapping, the generated values are more concentrated near 0 and 1 and less likely in the middle. Meanwhile, the distribution of the point cloud in Fig 2d shows obvious boundary aggregation, especially near the ends (such as 0.1 and 0.9) appearing high density of concentrated regions. The histogram of Sinusoidal chaotic mapping shows a non-uniform distribution, with a frequency number as high as 700 between [0.9, 1], which appears a higher concentration, as in Fig 2b. The scatter plot of the Sinusoidal map in Fig 2e also shows a certain boundary aggregation phenomenon, with concentrated values of [0, 0.1] and [0.9, 1], and a partial blank and loosely-distributed distribution between [0.6, 0.8]. In view of this, a new mutation chaotic strategy is proposed in this paper to make up for the shortcomings of Logistic and Sinusoidal chaotic map, and then improve the lack of diversity and uneven distribution of the original population of NSGA-II. The expression of the mutation chaos strategy is given in Eq. 11. In the formula, $\beta_{t_{1}},\sigma_{t_{2}}$ is the original sine mapping, *mod* is the complementary function.

$$\begin{array}{c} \begin{array}{cc} & \beta_{t1}=4\cdot \beta_{t1-1}\cdot (1-\beta_{t1-1})\\ & \sigma_{t2}=0.99\cdot \sin(\pi \cdot \sigma_{t2-1})\\ & \kappa_{t}=\mathrm{mod}(\beta_{t_{1}}+\sigma_{t_{2}},1) \end{array} \end{array}$$ (11)

In Fig 2c, the histogram distribution of the mutated chaotic strategy is more uniform, and the frequency is concentrated around 250. Compared with the Logistic and Sinusoidal mapping, there is no obvious concentrated area. From Fig 2f, it is found that the mutation chaos strategy has better uniformly distributed random values, and the boundary aggregation phenomenon disappears, making the initialized individuals random and uniform.

#### 3.2.3 Dynamic goal-oriented development.

NSGA-II individuals evolve through continuous cross and mutation to maintain population diversity. Given adequate time, satisfactory solutions will always be obtained by preserving superior individuals. Therefore, NSGA-II is greatly affected by the number of iterations, and there is a timeliness problem. This paper proposes a dynamic goal-oriented development method to accelerate the evolution of NSGA-II individuals to the Pareto front. Here, a dynamic adjustment factor *r* is used, which not only ensures the population diversity of crossover and mutation, but also uses goal-directed development to speed up the convergence of the algorithm. In the formula, *Ms* is a custom constant.


$$\begin{array}{c} \begin{array}{cc} & r_{c}=M_{S}-(\frac{M_{S}\cdot Iter}{Max-iter})\\ & r=2\cdot r_{c}\cdot rand-r_{c} \end{array} \end{array}$$ (12)

**Fig 3:**
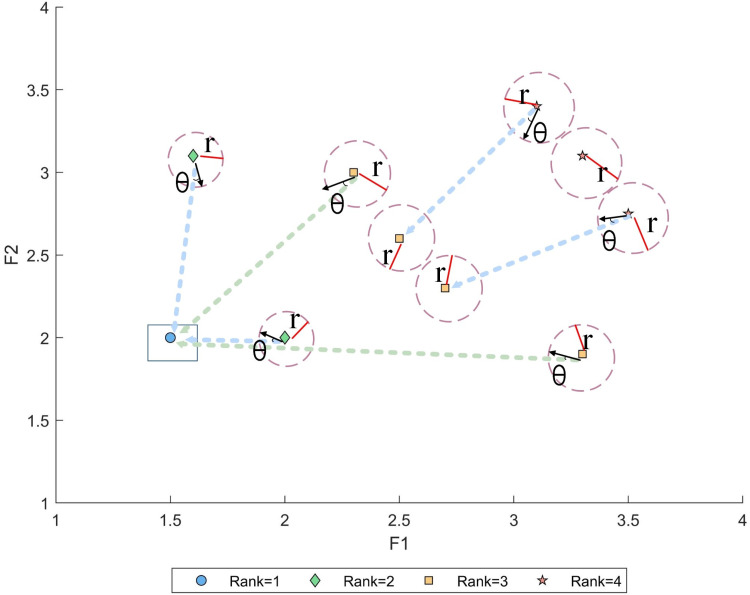
Dynamic goal-oriented development strategy.


As shown in Fig 3, after the non-dominated sorting and crowding degree calculation of individuals, the second-ranked individual with the largest crowding degree begins to evolve toward the first-ranked individual by generating a random angle from the connection with the target point. Subsequently, the following individuals evolve towards higher-ranked individuals, thereby reducing the algorithm’s calculation time and iteration count.

$$\begin{array}{c} \begin{array}{cc} & \beta_{s}=\left|rand\cdot \beta_{best}(t)-\beta (t)\right|\\ & \beta (t+1)=\beta_{best}(t)-r_{c}\cdot rand\cdot \beta_{s}\cdot \cos(\theta ) \end{array} \end{array}$$ (13)

Among them, $\beta_{best}(t)$ is the top-ranked individual of the current iteration, β(t) is the current low-ranked individual with large crowding degree, and *θ* is the angle randomly selected by roulette wheel.

**Fig 4:**
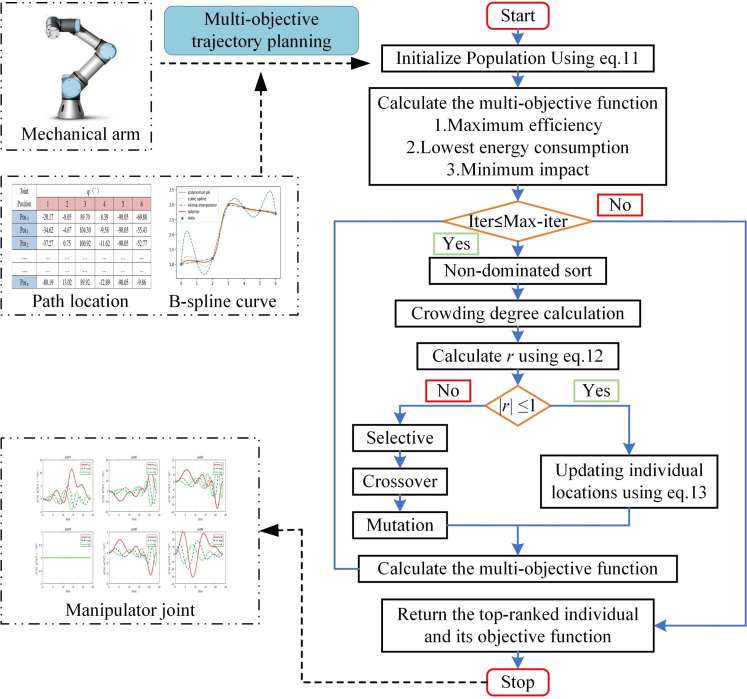
Trajectory planning model based on LNSGA-II.

In summary, firstly, the constraint problem is solved by designing the infeasible degree, then the mutation sine chaotic map is used to initialize the population individual, and finally the dynamic goal-oriented development is used to accelerate the iterative convergence of the individual, forming a new LNSGA-II algorithm. The specific process of applying LNSGA-II to solve the multi-objective trajectory optimization problem is shown in Fig 4.

Fig 4 depicts a multi-objective trajectory planning model for the manipulator. Firstly, the time and sequence of the key nodes of the motion path are calculated by APF in 2.1 to model the manipulator’s trajectory. Then, the LNSGA-II individual is initialized, and the time of the control node of the 7-order B-splines curve is taken as the independent variable. The optimization of ‘efficiency-energy-impact’ is defined as the multi-objective function, initiating iterative calculations until the termination condition is met. Finally, the time of the control node is output to complete the multi-objective trajectory planning of the manipulator.

## 4. Numerical experiment and trajectory planning experiment

### 4.1 Numerical experiment

In this paper, LNSGA-II is compared with MODE [40], MOGWO [41], MOPSO [42], NSGA-II and NSGA-III [43] through the test set CEC2009. The CEC2009 test set has been used by a wide range of scholars to test the numerical performance of multi-objective optimization algorithms [44,45], and the specific functions and code have been made publicly available at https://github.com/P-N-Suganthan/CEC2009-MOEA. Table 2 shows the function names of CEC2009.

**Table 2:** CEC2009 test set.

CEC2009 [46]	No	
Unconstrained Problem Function (UF)	1–7	Bi-objective function
	8–10	Triple objective function
Constrained Problem Function (CF)	1–7	Bi-objective function
	8–10	Triple objective function

The performance of the multi-objective algorithms is evaluated using Inversion Generational Distance (IGD) [47], Spacing (SP) [48] and Maximum Spread (MS) [49], which are mathematically expressed as follows:


$$\begin{array}{c} IGD=\frac{\sqrt{\underset{i=1}{\overset{k}{\sum }}\;d_{i}^{2}}}{n}\\ SP=\sqrt{\frac{1}{n-1}\times \underset{i=1}{\overset{k}{\sum }}\;{\left(\bar{d}-d_{i}\right)}^{2}}\\ MS=\sqrt{\underset{i=1}{\overset{H}{\sum }}\;\max(d(a_{i},b_{i}))} \end{array}$$ (14)

Where $\begin{array}{c} d_{i}=\min(\left|f_{1}^{i}(x)-f_{1}^{j}(x)\right|+\left|f_{2}^{i}(x)-f_{2}^{j}(x)\right|) \end{array}$, *i, j = 1,2..., f*_*1*_*, f*_*2*_ are the objective functions. *a*_*i*_ and *b*_*i*_ represent the maximum and minimum values of the ith objective, and *H* represents the number of objective functions. The experiment is carried out under Windows 11,64-bit operating system, and the MATLAB version is R2023a. The experimental parameters are set as Table 3 and the *N* is 30, the *max-iteration* is 200, the cycle is 30 times, and the variable dimension is 10.

**Table 3:** Experimental parameter settings.

Comparison algorithm	Parameter settings
MODE	Scaling factor = 0.5, Crossover probability = 0.2
MOGWO	β = 4, γ = 2
MOPSO	w = 0.4, c_1_ = c_2_ = 2
NSGA-II	Crossover = 0.9, mutation = 0.5
NSGA-III	Crossover = 0.5, mutation = 0.5
LNSGA-II	Crossover = 0.9, mutation = 0.5

**Fig 5:**
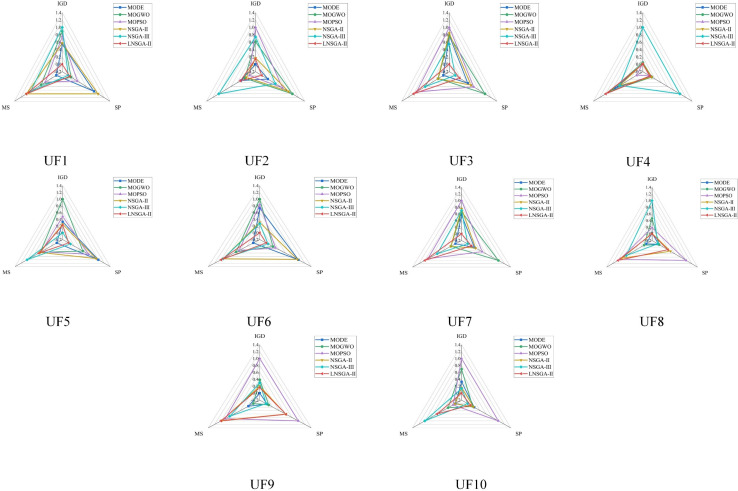
Visualization result of IGD, SP and MS on UF1-10.

Tables 4–6 is the statistical data of LNSGA-II and MODE, MOGWO, MOPSO, NSGA-II, NSGA-III in UF1–10, including the three evaluation indexes of IGD, SP, and MS. It can be seen from the table that LNSGA-II occupies the first place in IGD and MS, and only lags behind NSGA-III in SP. For example, the IGD of LNSGA-II in UF3 is 0.2690, which is less than 0.3803 of NSGA-III, and the average value ranks first. However, its standard deviation of 0.0461 is larger than that of 0.0411 of NSGA-III, and it ranks the second, and the difference between the two is small. In UF8, the average SP value of NSGA-III is 0.0862 and the standard deviation is 0.0282, which is far more than 0.4754 and 0.2709 of LNSGA-II, and then ranks first. Fig 5 is the visualization result of the normalized statistical data of the above Tables 4–6, in which IGD and SP are the smaller the better, while MS is the opposite.

**Table 4:** Statistical data of the IGD on UF1-10.

IGD	UF1						UF2					
	MODE	MOGWO	MOPSO	NSGA-II	NSGA-III	LNSGA-II	MODE	MOGWO	MOPSO	NSGA-II	NSGA-III	LNSGA-II
Mean	0.0837	0.1106	0.0997	0.0859	0.1191	**0.0418**	**0.0257**	0.0546	0.0728	0.0337	0.0606	0.0324
Std	0.0243	0.0192	0.0080	0.0276	0.0456	**0.0035**	**0.0030**	0.0068	0.0083	0.0093	0.0323	0.0069
	UF3						UF4					
Mean	0.4281	0.4268	0.4722	0.4400	0.3803	**0.2690**	0.0502	0.0732	0.0571	0.0508	0.6644	**0.0426**
Std	0.0547	0.0889	0.1630	0.0660	**0.0411**	0.0461	0.0038	0.0077	0.0039	0.0043	0.5910	**0.0021**
	UF5						UF6					
Mean	0.6392	1.1344	0.7547	0.5862	**0.3984**	0.5640	0.8649	1.0425	0.9334	0.5901	0.5635	**0.3864**
Std	**0.0624**	0.3179	0.2652	0.2121	0.0924	0.1671	0.1004	0.3300	0.1739	**0.0912**	0.1184	0.1168
	UF7						UF8					
Mean	0.3947	0.4140	0.4802	0.4323	0.4220	**0.2831**	0.2178	0.4758	0.3122	0.2093	0.7945	**0.2010**
Std	0.0538	0.0790	0.1533	0.0683	0.0562	**0.0401**	0.0552	0.4035	0.0455	0.0326	0.2989	**0.0155**
	UF9						UF10					
Mean	**0.1899**	0.2887	0.4451	0.2411	0.2658	0.2304	1.2792	2.1296	2.8343	0.8819	0.8328	**0.5209**
Std	**0.0219**	0.0868	0.0878	0.0765	0.0848	0.0494	0.2335	0.5858	0.7249	0.3131	0.1867	**0.0623**
Rank	2	6	5	4	3	1						

**Table 5:** Statistical data of the SP on UF1-10.

SP	UF1						UF2					
	MODE	MOGWO	MOPSO	NSGA-II	NSGA-III	LNSGA-II	MODE	MOGWO	MOPSO	NSGA-II	NSGA-III	LNSGA-II
Mean	0.2255	0.0463	0.0951	0.2574	**0.0241**	0.0366	0.0249	0.0663	0.0497	0.0598	0.0376	**0.0146**
Std	0.0635	0.0429	0.0924	0.0898	0.0300	**0.0098**	0.0095	0.0294	0.0324	0.0194	0.0215	**0.0056**
	UF3						UF4					
Mean	0.1218	0.2081	0.1518	0.1375	**0.0588**	0.0803	0.0207	0.0290	**0.0122**	0.0279	0.1949	0.0178
Std	0.0736	0.2423	0.1665	0.0675	0.0607	**0.0164**	0.0050	0.0035	**0.0029**	0.0038	0.1088	0.0053
	UF5						UF6					
Mean	0.4880	0.2962	0.3678	0.4618	0.1418	**0.1192**	0.8242	0.2511	0.3100	0.8045	0.1396	**0.0982**
Std	0.1590	0.2256	0.2538	0.1393	**0.0342**	0.1150	0.3346	0.1701	0.3153	0.2901	**0.0402**	0.1076
	UF7						UF8					
Mean	0.1057	0.2865	0.1638	0.1145	**0.0552**	0.0829	0.1235	0.1445	1.1833	0.5743	**0.0862**	0.4754
Std	0.0372	0.3002	0.1600	0.0406	0.0678	**0.0151**	0.0608	0.0763	0.6042	0.5638	**0.0282**	0.2709
	UF9						UF10					
Mean	0.1163	0.1713	1.2314	0.7822	**0.0795**	0.7899	0.9300	0.7422	3.8322	0.8514	**0.1559**	0.4822
Std	**0.0320**	0.0813	0.5506	0.4350	0.0653	0.5000	0.9123	0.2368	1.9838	0.6286	**0.1234**	0.1309
Rank	4	5	3	6	1	2						

**Table 6:** Statistical data of the MS on UF1-10.

MS	UF1						UF2					
	MODE	MOGWO	MOPSO	NSGA-II	NSGA-III	LNSGA-II	MODE	MOGWO	MOPSO	NSGA-II	NSGA-III	LNSGA-II
Mean	0.6549	0.9538	0.8707	**1.2447**	1.1632	1.2430	0.0249	0.7791	0.7556	0.7263	0.7458	**0.9162**
Std	0.0813	0.1787	**0.1900**	0.0885	0.1820	0.0672	0.0095	0.0941	0.0955	**0.1509**	0.0869	0.1094
	UF3						UF4					
Mean	0.5608	0.7320	1.4599	0.7435	1.1702	**1.5576**	0.7350	0.8181	0.3469	0.6654	0.6555	**0.9086**
Std	0.0630	0.0820	0.3971	0.0746	0.1380	**0.4430**	0.0802	0.1338	0.0486	0.0633	0.0530	**0.1471**
	UF5						UF6					
Mean	0.6231	0.9262	0.9049	0.9264	**1.1645**	0.9518	0.6669	0.9131	0.8725	1.1004	1.1006	**1.1289**
Std	0.0599	0.1463	**0.1785**	0.0819	0.1448	0.0790	0.0586	0.1500	0.1391	0.0993	0.1056	**0.1539**
	UF7						UF8					
Mean	0.5742	0.7156	1.4416	0.7207	1.1488	**1.5383**	0.6247	0.6511	0.9230	0.9036	0.8518	**0.9466**
Std	0.0633	0.0595	0.3591	0.0616	0.1468	**0.4247**	0.0634	0.0903	0.1795	0.1608	0.0859	**0.1866**
	UF9						UF10					
Mean	0.6564	0.6020	0.9163	0.9542	0.8669	**0.9634**	0.5570	0.6741	0.5951	0.5638	**1.0465**	0.8505
Std	0.0703	0.0884	**0.1654**	0.1266	0.1359	0.1097	0.0688	0.1075	0.1315	0.1073	0.0911	**0.1591**
Rank	6	5	2	4	3	1						

**Fig 6:**
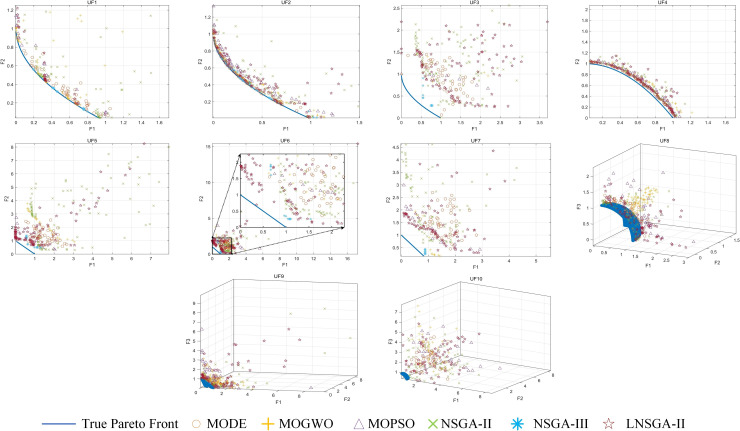
Pareto solutions for UF1-10.

Fig 6 shows the set of pareto solutions of the six algorithms on UF1–10. The figure shows that compared with NSGA-II, the solution set of LNSGA-II is closer to the true pareto frontier and lags behind NSGA-III, which illustrates the competitiveness and advantage of LNSGA-II in solving unconstrained optimization problems.

**Fig 7:**
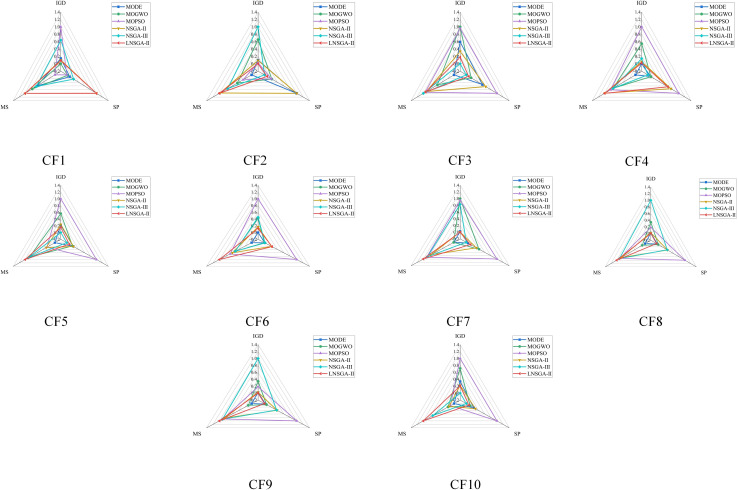
Visualization result of IGD, SP and MS on CF1-10.

Tables 7–9 shows the statistical data of CF1-10 of LNSGA-II and MODE, MOGWO, MOPSO, NSGA-II and NSGA-III in CEC2009, including the mean value and standard deviation of the three evaluation indicators of IGD, SP and MS. The normalized visualization results are shown in Fig 7. In CF6, the MS of LNSGA-II is 1.1707, ranking first, far exceeding the 0.6648 of MODE. The IGD of NSGA-III in CF10 is 0.5135, which is more than 0.9630 of LNSGA-II, ranking first. At the same time, MOPSO’s 2.6770 ranks last. In general, NSGA-III ranks first in the CF1-10 solving process, while LNSGA-II is followed.

**Table 7:** Statistical data of the IGD on CF1-10.

IGD	CF1						CF2					
	MODE	MOGWO	MOPSO	NSGA-II	NSGA-III	LNSGA-II	MODE	MOGWO	MOPSO	NSGA-II	NSGA-III	LNSGA-II
Mean	0.0165	**0.0149**	0.0260	0.0160	0.0220	0.0158	0.0997	0.1611	0.0922	0.0989	0.1993	**0.0890**
Std	0.0035	0.0031	0.0019	**0.0012**	0.0039	0.0013	0.0380	0.0363	**0.0115**	0.0499	0.0958	0.0262
	CF3						CF4					
Mean	1.0416	1.4533	1.4076	0.7843	**0.4465**	0.6237	**0.1206**	0.2960	0.4430	0.1392	0.1657	0.1271
Std	0.1168	0.6427	0.3139	0.1370	**0.0777**	0.2519	**0.0132**	0.2439	0.1688	0.0423	0.0588	0.0330
	CF5						CF6					
Mean	0.8616	1.7447	2.8608	0.9253	**0.3074**	0.6838	**0.0368**	0.1238	0.2417	0.0718	0.1301	0.0617
Std	0.3256	0.6620	0.9192	0.2849	**0.0730**	0.1367	**0.0058**	0.0510	0.0676	0.0207	0.0640	0.0111
	CF7						CF8					
Mean	1.1241	2.7178	2.9203	0.6411	**0.3331**	0.6110	0.2720	0.7376	0.7129	0.2611	0.6558	**0.2594**
Std	0.2464	0.8117	1.0566	0.1942	**0.1002**	0.1739	0.0366	0.8995	0.1920	0.0483	0.3324	**0.0326**
	CF9						CF10					
Mean	0.2354	0.4436	0.3423	0.2378	0.8721	**0.2209**	1.2419	2.0560	2.6770	0.9997	**0.5135**	0.9630
Std	0.0392	0.2587	0.0626	0.0318	0.3062	**0.0188**	0.2315	0.5706	0.6037	0.3805	**0.0948**	0.2607
Rank	3	5	4	6	1	2						

**Table 8:** Statistical data of the SP on CF1-10.

SP	CF1						CF2					
	MODE	MOGWO	MOPSO	NSGA-II	NSGA-III	LNSGA-II	MODE	MOGWO	MOPSO	NSGA-II	NSGA-III	LNSGA-II
Mean	0.0208	0.0130	**0.0102**	0.1173	0.0349	0.1179	0.2723	0.0750	0.0715	0.2747	**0.0168**	0.0376
Std	0.0053	0.0026	**0.0014**	0.0282	0.0142	0.0404	0.0681	0.1496	0.0494	0.0839	0.0202	**0.0125**
	CF3						CF4					
Mean	0.8548	0.3199	1.5455	1.0140	**0.0936**	0.2061	0.0899	0.0948	0.8473	0.6359	**0.0134**	0.5527
Std	0.2431	0.2787	1.4758	0.3702	**0.1021**	0.1021	0.0523	0.0759	0.6722	0.2562	**0.0258**	0.1836
	CF5						CF6					
Mean	0.5174	0.4663	2.2951	0.5449	**0.0328**	0.2273	0.0396	**0.0328**	0.7128	0.1812	0.0426	0.1912
Std	0.1571	0.4905	2.2744	0.2058	**0.0728**	0.0733	**0.0117**	0.0269	0.6586	0.1046	0.0318	0.0963
	CF7						CF8					
Mean	1.0564	0.8127	1.9341	1.1054	0.2828	**0.1162**	0.2247	1.0184	2.3323	0.8309	**0.0895**	0.3935
Std	0.3398	0.7263	1.5152	0.3769	**0.0585**	0.2145	0.0869	1.0064	1.0267	0.6567	**0.0540**	0.2705
	CF9						CF10					
Mean	0.1156	0.1455	1.0037	0.4441	0.4325	**0.0888**	1.0576	0.6125	3.7814	1.2329	**0.1581**	0.4711
Std	**0.0275**	0.0888	0.5505	0.4093	0.3069	0.0422	0.8035	0.2520	1.4921	1.3880	**0.1023**	0.1036
Rank	3	5	4	6	1	2						

**Table 9:** Statistical data of the MS on CF1-10.

MS	CF1						CF2					
	MODE	MOGWO	MOPSO	NSGA-II	NSGA-III	LNSGA-II	MODE	MOGWO	MOPSO	NSGA-II	NSGA-III	LNSGA-II
Mean	0.7075	0.8258	0.2559	1.0039	0.7249	**1.0045**	0.6701	0.9352	0.8294	1.2589	1.1147	**1.2703**
Std	0.0715	**0.1501**	0.0425	0.0816	0.0947	0.0967	0.0804	**0.1907**	0.1490	0.0909	0.1377	0.0848
	CF3						CF4					
Mean	0.7546	0.9583	1.1093	1.0947	**1.1320**	1.1037	0.6312	1.0615	1.1130	1.2206	1.0692	**1.2288**
Std	0.0770	0.1660	**0.2931**	0.0989	0.1472	0.0983	0.1119	0.1947	**0.2698**	0.1135	0.0984	0.1060
	CF5						CF6					
Mean	0.6386	1.0045	0.7531	0.7628	1.0218	**1.0666**	0.6648	0.9242	1.0031	0.9667	0.9205	**1.1707**
Std	0.0759	0.1625	0.0594	0.0605	**0.2684**	0.1315	0.0981	0.1463	0.0999	0.1035	0.1243	**0.2760**
	CF7						CF8					
Mean	0.6397	0.9130	0.9176	0.9130	**1.1947**	1.0025	0.6723	0.6752	0.8906	0.8564	0.8829	**0.9164**
Std	0.0542	0.1687	0.0744	0.0827	**0.2460**	0.2163	0.0898	0.1194	**0.1770**	0.1399	0.0984	0.1738
	CF9						CF10					
Mean	0.6348	0.6631	0.8770	0.8646	0.8810	**0.9079**	0.5316	0.6307	0.5903	0.6135	0.8802	**1.0353**
Std	0.0598	0.0798	0.1902	**0.1955**	0.0761	0.1619	0.0429	0.0848	0.1099	**0.1657**	0.1341	0.1130
Rank	6	5	3	4	2	1						

**Fig 8:**
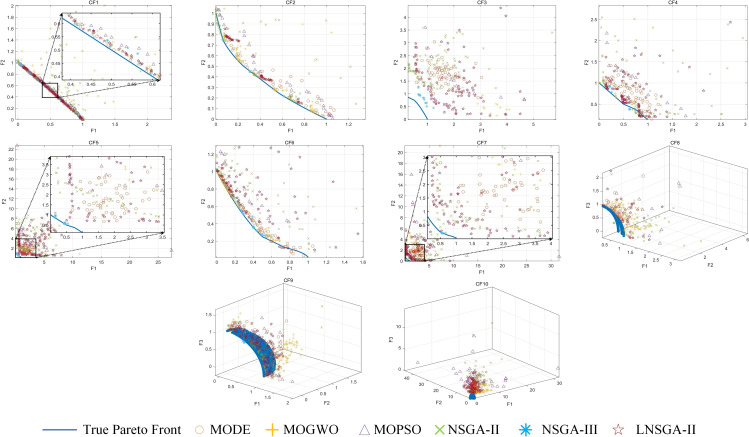
Pareto solutions for CF1-10.

Fig 8 is the Pareto solution set of the above algorithm in CF1–10. It can be seen from the figure that LNSGA-II is obviously better than NSGA-II, and it is closer to the real Pareto frontier under the same experimental conditions, indicating the effectiveness and success of the improved strategy in this paper. NSGA-III is superior to LNSGA-II only by a slight advantage in the evaluation index, which highlights that LNSGA-II is highly competitive in constrained multi-objective optimization problems.

### 4.2 Trajectory planning experiment

In order to meet the actual operation requirements of the manipulator, trajectory planning needs to ensure fast motion while avoiding the wear of the manipulator caused by motion impact and the reduction of trajectory accuracy. Accordingly, the shortest movement time, the minimum energy consumption of the overall joint and the smooth control in the motion performance of the manipulator are taken as the optimization objectives. The 7-order B-splines curve is used to interpolate the trajectory to ensure the high-order continuity of the manipulator trajectory and improve the smoothness of the trajectory. Simultaneously, a multi-objective optimization algorithm is applied to achieve a well-distributed set of Pareto solutions.

Taking the 6-DOF robotic arm as the experimental object, the sequence of joint positions is obtained through 2.1, as shown in Table 1. Among them, the kinematic and torque constraints of each joint are shown in Table 10. In the table, $v_{\max},a_{\max},j_{\max},\tau_{\max}$ are the maximum velocity, acceleration, jerk and torque of the joint respectively.

**Table 10:** Joint constraints of the 6-DOF robotic arm.

NO. Joint	v_max_ ((^。^)∙s^-1^)	a_max_ ((^。^)∙s^-2^)	J_max_ ((^。^)∙s^-3^)	T_max_ (N ∙ m)
1	120	45	90	327
2	120	40	80	167
3	180	75	70	167
4	180	70	55	20
5	180	90	60	10
6	180	80	60	10

**Fig 9:**
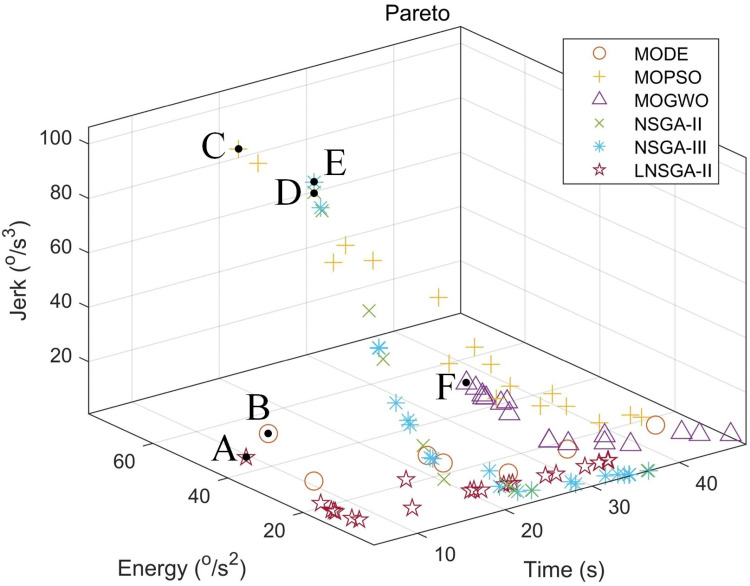
Pareto solution set for multi-objective trajectory planning.

Fig 9 shows the Pareto front of the multi-objective trajectory planning of the manipulator joint. The optimal energy consumption and impact optimal performance are in the same direction, which is opposite to the efficiency optimal performance. The optimization results of the six points of A-F are shown in Table 11.

**Table 11:** Optimization results of Pareto frontier points.

Multi-objective optimization algorithm	Optimal Results	f_1_ (s)	f_2_ ((^。^)∙s^-2^)	f_3_ ((^。^)∙s^-3^)
LNSGA-II	A	5.003	31.1462	6.4466
MODE	B	7.0699	33.9914	19.1328
MOPSO	C	22.1769	77.1118	83.2122
NSGA-II	D	12.6863	34.8764	102.2870
NSGA-III	E	12.7176	33.2282	97.7038
MOGWO	F	25.1574	23.2782	28.8125

In Fig 9, the Pareto front of LNSGA-II is significantly better than other algorithms, and better efficiency, energy consumption and impact are obtained. Table 12 is the node vector of the B-splines curve after the optimal control of the five algorithms at the A-F frontier point.

**Table 12:** Node variables after Pareto front point optimization.

	Vector of time nodes of the joint trajectories	The nodal vector of the B-spline curve
MODE	Δt=[0.29,2.60,4.25,0.34,7.46,2.50,3.94]	t=[0,0,0,0,0,0,0,0,0.0135,0.1353,0.3339,0.3498,0.6989,0.8157,1,1,1,1,1,1,1,1]
MOGWO	Δt=[3.04,2.76,1.02,0.26,0.04,0.08,0.07]	t=[0,0,0,0,0,0,0,0,0.4177,0.7971,0.9375,0.9375,0.9794,0.9902,1,1,1,1,1,1,1,1]
MOPSO	Δt=[5.49,4.68,4.60,3.13,2.80,3.45,4.89]	t=[0,0,0,0,0,0,0,0,0.2509,0.4647,0.5155,0.6010,0.7570,0.7962,1,1,1,1,1,1,1,1]
NSGA-II	Δt=[0.29,2.60,4.25,0.34,7.46,2.50,3.94]	t=[0,0,0,0,0,0,0,0,0.2069,0.3494,0.5078,0.6159,0.7123,0.8314,1,1,1,1,1,1,1,1]
NSGA-III	Δt=[3.17,1.12,0.66,2.09,0.03,0.02,0.06]	t=[0,0,0,0,0,0,0,0,0.4433,0.5998,0.6921,0.9847,0.9893,0.9917,1,1,1,1,1,1,1,1]
LNSGA-II	Δt=[3.4,0.6,2.75,1.3,2.1,0.85,1.6]	t=[0,0,0,0,0,0,0,0,0.0135,0.1353,0.3339,0.3498,0.6989,0.8157,1,1,1,1,1,1,1,1]

**Fig 10:**
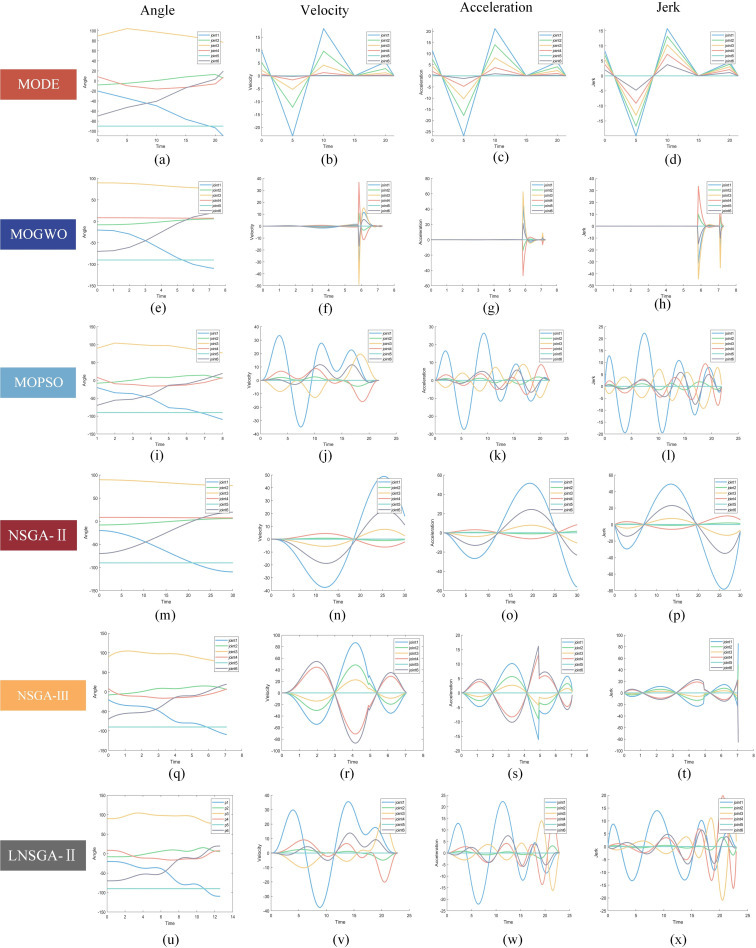
Experimental results of multi-objective trajectory planning.

Fig 10 shows the multi-objective trajectory planning results of the six algorithms at six points A-F on the Pareto front, showing the optimized angle, velocity, acceleration and jerk curves, respectively. The following conclusions can be drawn from the figure:

(1) From the angle curve of the first column, (a) and (i) indicates that MODE and MOPSO exhibit insufficient smoothness in their angle curves. There are inflection points in the motion angle, which will cause jitter during the motion of the manipulator. The angle curves of the other three algorithms are smooth and continuous.
(2) In the velocity, acceleration and jerk curves of columns 2–4, MOPSO, NSGA-II and LNSGA-II all meet the basic requirements of manipulator joint trajectory planning: uniform, smooth and continuous. Despite meeting the basic criteria, MOPSO and NSGA-II lag significantly behind LNSGA-II in multi-objective function optimization, impacting the precise attainment of the ‘efficiency-energy-impact’ goal.
(3) From Figures (b)-(d), MODE exhibits sharp corners and sudden changes in velocity, acceleration, and jerk curves at inflection points, potentially inducing undesirable impact fluctuation and motor damage.
(4) Analysis of Figures (f)-(h) demonstrates that MOGWO initially experiences minimal fluctuations in speed, acceleration, and jerk during trajectory planning. However, sharp curve fluctuations at 6s result in abrupt changes impacting the manipulator’s speed, acceleration, and jerk. This is derived from the fact that when MOGWO optimizes the node variables of the B-spline curve, the subsequent time nodes are too compact and change less.
(5) Figures (r)-(t) show that the velocity and acceleration curve of NSGA-III change dramatically at 5s, because Δt is 0.03 and the change is too small. Similarly, the jerk curve of the 7s shows sharp changes.

Trajectory planning involves using a 7-order B-splines curve, with LNSGA-II adopted for multi-objective optimization of the B-spline curve node vector. The velocity, acceleration and jerk of each joint of the manipulator in the start and stop stages are 0. The overall joint motion trajectory is smooth and continuous, which reduces the vibration of the manipulator and effectively protects the key components such as motor and reducer.

**Fig 11:**
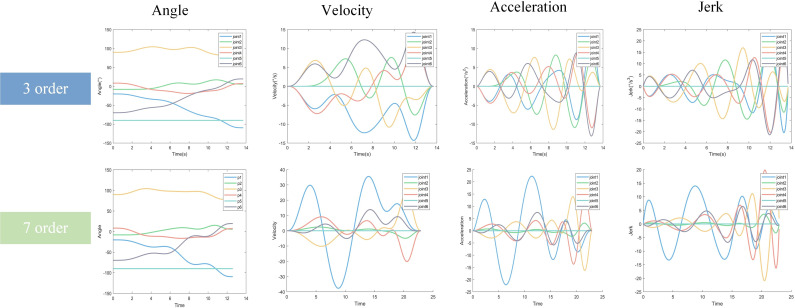
Comparative experiments on spline curves.

Taking the time of the control node of the 7th-order B-spline curve as the optimization variable, this paper adopts the LNSGA-II algorithm to solve the multi-objective function of “optimal efficiency, energy consumption, and impact”, and completes the multi-objective trajectory planning of the robotic arm. The experimental results show that compared with similar optimization algorithms, the LNSGA-II algorithm is more successful. In this paper, the order of the B-spline curve is chosen because in the same experimental platform, the computation time of the B-spline curves of order 3, 5 and 7 are in the same order of magnitude, so a higher order is chosen. To ensure the reliability of the study, multi-objective trajectory planning experiments were conducted using the LNSGA-II algorithm to optimize the B-spline curves of orders 3 and 7, respectively. Fig 11 shows the comparison experiments of 3rd and 7th order B-spline curves.

As can be seen from Fig 11, the third-order B-spline curve obviously reduces the angular velocity, angular acceleration, and jerk of joint 1, but leads to sharp changes in the values related to angular velocity, angular acceleration, and jerk of joints 2 and 4, which may be the result of the trade-off between the third-order B-spline and the seventh-order B-spline under different objective constraints. Table 13 shows a comparison of the Pareto solution set results for two different orders of B-spline curves (bold indicates the best result).

**Table 13:** Comparison of Pareto solution set results.

	Number of Pareto solution sets	Time (s)		Energy consumption (^。^/s^2^)		Impact (^。^/s^3^)		Running time (s)
		Minimum value	Maximum value	Minimum value	Maximum value	Minimum value	Maximum value	
Third order B-splines	21	13.7080	36.7325	**1.0446**	**27.9954**	**0.8048**	68.4718	3046
Seventh-order B-splines	23	**5.0035**	**33.4127**	4.6446	35.1462	5.0506	**11.4466**	3387

The running time of the third-order B-spline curve is 3046s, which is smaller than the 3387s of the seventh-order B-spline, which is obvious that the increase in the order of the B-spline curve inevitably increases the computation time of the algorithm, and the difference between the two running times is extremely small. Despite the increase in computation time, the seventh-order B spline curve obtains more Pareto solution set results. At the same time, in the three evaluation indexes of multi-objective trajectory planning, the values of time and impact of the seventh-order B spline curve are smaller than those of the third-order B spline curve, and only in the energy consumption indexes, it lags behind that of the third-order B spline curve, but the gap is smaller. Through the qualitative analysis of trajectory planning experiments, as well as the quantitative analysis of numerical indexes, this paper chooses the seventh-order B spline curve, by sacrificing part of the computation time, but obtains a better performance effect in multi-objective trajectory planning of the robotic arm, which is worthwhile.

## 5. Digital twin-based UR16e grasping task

### 5.1 Twin model of the robotic arm UR16e

The concept of digital twin was introduced by GRIEVES at the University of Michigan, then known as ‘Mirrored Spaces Model’ (MSM) [50], which defined as an object and its digitized mirror image and the connection between the two, and later in the literature as ‘digital twin’ [51].

**Fig 12:**
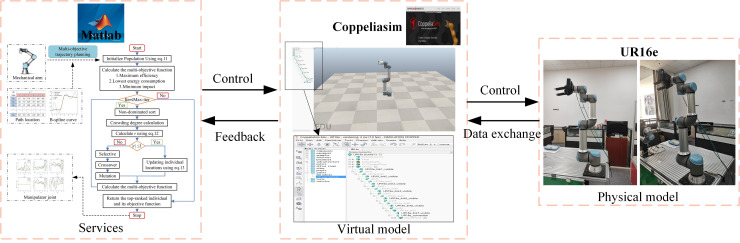
Digital twin of the UR16e.

A three-dimensional virtual model of UR16e robotic arm is established in this paper. Combined with physical entities, a digital twin model interaction platform is constructed through MATLAB-CoppeliaSim-UR16e, and trajectory planning model is applied. It solves the problems of large-scale rapid and disorderly loading process of the robotic arm in the large-scale start-stop impact, easy to jitter, and reduces the efficiency of the production operation and service life, and so on. The digital twin platform construction process is shown in Fig 12.

### 5.2 The grasping experiment of UR16e

**Fig 13:**
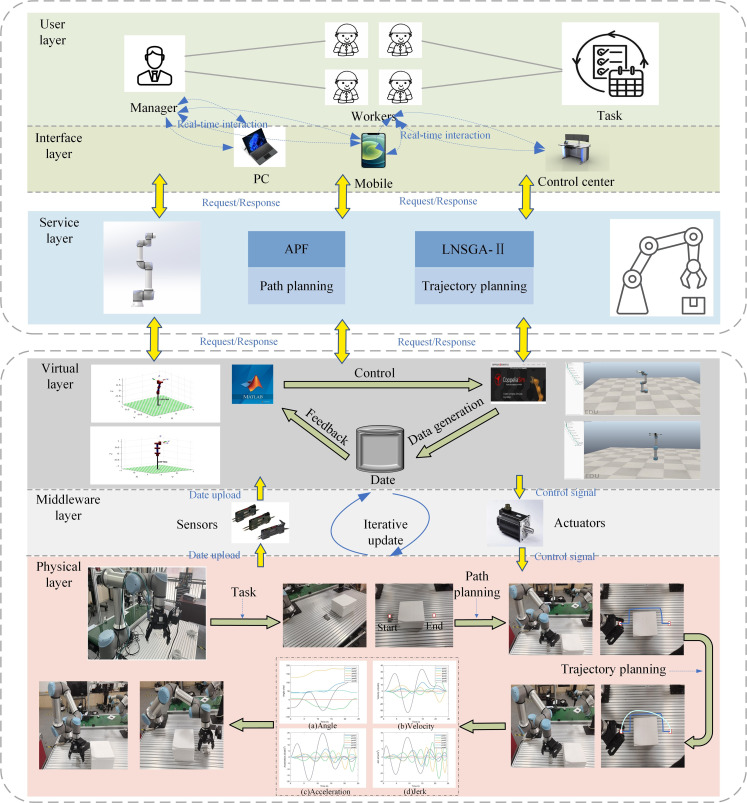
The digital twin frame of the robotic arm UR16e grasping the material.

The digital twin model framework of UR16e grasping material is shown in Fig 13. The framework consists of user layer, service layer, twin model layer and physical entity layer. Initially, when the user layer receives the robotic arm grasping task, the task instructions (the starting and ending coordinates of the material, the transportation time, etc.) are transmitted through the portable controller to the service layer. Subsequently, the service layer processes the signal, utilizes the APF algorithm to determine a collision-free shortest path for grasping, identifies key motion sequences, and solves the multi-objective motion trajectory (including angle, velocity, acceleration, and jerk motion curves for each joint) through the trajectory planning model. Then the twin model layer obtains the multi-objective trajectory of the grasping material and assesses the feasibility of the motion command through virtual model simulation. Finally, the motion command is sent to the physical entity layer to direct the manipulator in successfully completing the grasping task.

**Fig 14:**
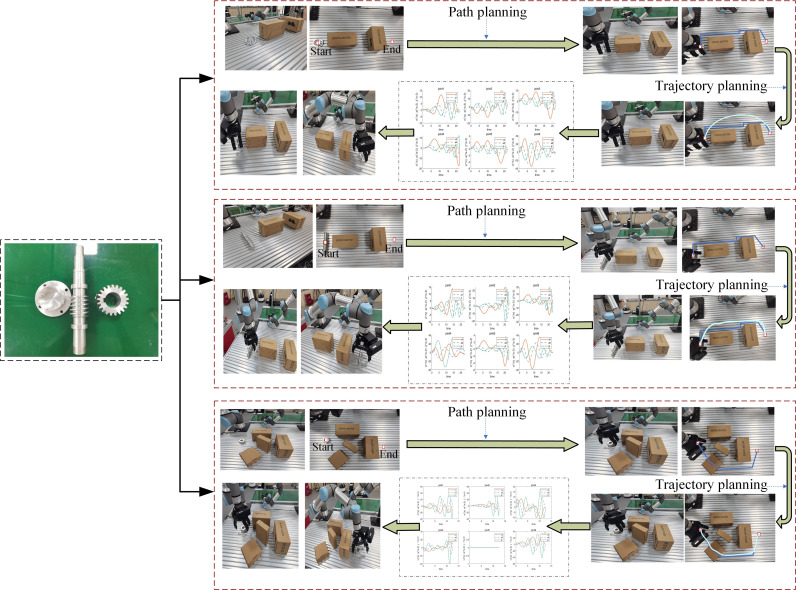
Process of grasping material experiment.

In order to verify the accuracy and reliability of the digital twin framework of UR16e grasping materials proposed in this chapter, three different mechanical parts (cover, worm, helical gear) are set up in this section with grasping experiments conducted in a real environment. The grasping process is shown in Fig 14, where the robotic arm UR16e sequentially grasps the three types of workpieces. The specific experimental results are presented in Table 14, including the number of successful multi-objective trajectory planning for each experimental group and whether they effectively reached the target point.

**Table 14:** Experimental results of grasping materials.

		Number of experiments	The number of successful multi-objective trajectory planning	Planning success rate	The number of successful grasps	Success rate
Group 1	Cover	20	20	100%	20	100%
Group 2	Worm	20	19	95%	18	90%
Group 3	Helical gear	20	18	90%	17	85%
Total		60	57	95%	55	91.7%

From the table, the following conclusions can be drawn:

(1) The overall success rate for multi-objective trajectory planning across the three experimental groups is 95%, with a grasping success rate of 91.7%, affirming the feasibility and effectiveness of the inverse solution optimization model and trajectory planning model in practical applications.
(2) Group 1 demonstrates higher success rates in trajectory planning and grasping for the cover due to its smaller volume and fewer environmental obstacles.
(3) Within Group 2, the trajectory planning success rate is 95%, indicating the successful resolution of trajectory motion curves for multiple targets with LNSGA-II. However, due to the length of the worm, instances of obstacle contact during grasping result in decreased success rates.
(4) In the third group of experiments, due to the increase of the complexity of obstacles, the success rate of multi-objective trajectory planning decreases, resulting in touching obstacles during the grasping process.

## 6. Conclusion

For the large-scale rapid and disorderly loading, the robotic arm has the problems of large start-stop impact, easy to shake, and reduced production efficiency and service life, etc. In this paper, the artificial potential field method is use to obtain the shortest collision-free gripping path, extract the key motion sequences, and use the B-spline curve to mathematically model the trajectory planning problem. The multi-objective function of “efficiency, energy consumption and impact optimization” is established. Secondly, considering the poor diversity of the initial population of NSGA-II and the problem of being greatly affected by the number of iterations, this paper proposes a new mutation chaos strategy and a dynamic goal-oriented development strategy. At the same time, aiming at the constraint problem of multi-objective trajectory planning of manipulator, an infeasible degree solution is designed, and a new multi-objective optimization algorithm LNSGA-II is formed. Through the CEC2009 test set experiment, compared with MODE, MOGWO, MOPSO, NSGA-II and NSGA-III, LNSGA-II has faster convergence speed and optimization ability, which shows the effectiveness and success of the improved strategy. Then, the time variable of the control node of the 7-node B-spline curve is used as the independent variable, and the LNSGA-II algorithm is utilized to solve the problem under the constraints of multi-objective function and joint conditions to complete the multi-objective trajectory planning in the joint space of the robotic arm. Finally, through MATLAB-CoppeliaSim-UR16e, the digital twin model of UR16e is built, and the above motion planning model is integrated, and then applied to the actual grasping task of UR16e after validation by the twin.

Although the LNSGA-II algorithm has a fast convergence speed, it still cannot meet the requirements of real-time motion planning for robotic arms, and real-time is one of its limitations. Future work will consider the deep learning approach for real-time motion planning of robotic arms. Meanwhile, this paper validates the application of the digital twin framework through a physical platform, but it is limited to the experimental environment. Applying it to real production operations will be a future research direction.
